# How Hinge Positioning in Cross-Country Ski Bindings Affect Exercise Efficiency, Cycle Characteristics and Muscle Coordination during Submaximal Roller Skiing

**DOI:** 10.1371/journal.pone.0153078

**Published:** 2016-05-20

**Authors:** Conor M. Bolger, Øyvind Sandbakk, Gertjan Ettema, Peter Federolf

**Affiliations:** 1 Center for Elite Sports Research, Norwegian University of Science and Technology, Trondheim, Norway; 2 Institute for Sports Sciences, University of Innsbruck, Innsbruck, Austria; University of Debrecen, HUNGARY

## Abstract

The purposes of the current study were to 1) test if the hinge position in the binding of skating skis has an effect on gross efficiency or cycle characteristics and 2) investigate whether hinge positioning affects synergistic components of the muscle activation in six lower leg muscles. Eleven male skiers performed three 4-min sessions at moderate intensity while cross-country ski-skating and using a klapskate binding. Three different positions were tested for the binding’s hinge, ranging from the front of the first distal phalange to the metatarsal-phalangeal joint. Gross efficiency and cycle characteristics were determined, and the electromyographic (EMG) signals of six lower limb muscles were collected. EMG signals were wavelet transformed, normalized, joined into a multi-dimensional vector, and submitted to a principle component analysis (PCA). Our results did not reveal any changes to gross efficiency or cycle characteristics when altering the hinge position. However, our EMG analysis found small but significant effects of hinge positioning on muscle coordinative patterns (P < 0.05). The changed patterns in muscle activation are in alignment with previously described mechanisms that explain the effects of hinge positioning in speed-skating klapskates. Finally, the within-subject results of the EMG analysis suggested that in addition to the between-subject effects, further forms of muscle coordination patterns appear to be employed by some, but not all participants.

## Introduction

In speed skating, the skate design underwent an important change with the introduction of the klapskate, in which the conventional rigid connection between the shoe and the blade was replaced by a hinge mechanism beneath the ball of the foot [1, 2]. The conventional system utilized a pivot point located in front of the foot, creating a long lever arm between the ankle joint and the line of action of the push-off (hinge position). This system was found to limit the plantar flexion, resulting in premature termination of the push-off. With the klapskate this lever arm was reduced, allowing for plantar flexion with the skate blade remaining flat whilst gliding on the ice and thereby increasing the effectiveness of the push-off [2, 3, 4]. As a result, skating velocity increased up to 5%, which was made possible due to an increase in mechanical power output generated across the ankle, knee and hip joints [2, 4]. These observed changes in kinematic and kinetic variables, leading to a higher mean power output, can be apprehended by increasing the work delivery by muscle-tendons as well as by a higher efficiency in the transformation of metabolic energy to power output [2]. This was later exemplified by an increase in gross efficiency [3].

With the development of the klapskate, it was found that changing the pivot position alters the push-off mechanics; specifically, it alters the lengths of the lever arms and thus the entire mechanical system of the lower extremity [1, 5]. For example, the long lever arm in conventional speed skates inhibited the athletes’ ability to initiate ankle rotation, which resulted not only in the suppression of plantar flexion, but also in the early termination of knee and hip extension [1, 2]. Furthermore, this spatial relationship between the pivot point and the ankle joint has also been studied in cycling with regard to shoe-pedal attachment locations, where moving the pivot position between the head of the first metatarsal and the posterior end of the calcaneus created a short lever arm, thereby reducing the ability of the ankle plantar flexors to generate power [6]. Another example where equipment modification affects the lever arm system can be found in sports such as in running. Here the ankle moment is constrained by the longitudinal bending stiffness of the sole and the effective pivot position changes based on the sole stiffness [7, 8].

The movement of cross-country ski-skating is similar to that of speed skating, as both utilize a push-off performed perpendicular to the skate/ski whilst gliding forward. Therefore, replacing the currently used flexible boot sole with a stiff sole and placing a hinge beneath the foot may not only be a good strategy to improve the effectiveness of the push-off in speed skating, but also in ski-skating [9].

Although the role of pivot positioning and the mechanisms involved have been studied extensively [2, 4, 5, 10], expected changes in the muscular coordination were neither found when comparing conventional skates and klapskates [1], nor in response to shifting the hinge placement. However, since clear changes in the movement between hinge positioning were found, differences in muscular activation must take place. Specifically, when hinge positioning affects the effective musculo-skeletal lever arms during the push-off, one would expect a change in the timing and amplitude of the muscles involved in the push-off (lateral gastrocnemius (GL), rectus femoris (RF), vastus medialis (VM), vastus lateralis (VL), tibialis anterior (TA)).

In spite of the fact that high variability is exhibited within electromyographic (EMG) signals among individuals, shared temporal features often reflect how muscles are active during a given movement [11]. Principle component analysis (PCA) can be applied to the EMG signals to detect patterns of muscle activation across a subset of muscles during a variety of motor tasks [11, 12, 13, 14]. We hypothesized that analyzing PCA patterns will provide insight into the muscle coordination patterns expected in push-off effectiveness while changing hinge position.

Therefore, the purposes of the current study were twofold: first, we hypothesized that in ski-skating—similar to speed skating—hinge positioning has an effect on gross efficiency and cycle characteristics. Second, we investigated whether hinge positioning affects synergistic components of the muscle activation in six lower leg muscles.

## Materials and Methods

### Participants

Eleven Norwegian national and international level skiers (male, age: 22–29; height: 1.80 ± 0.08 m; body mass: 75.5 ± 7.4 kg; International Ski Federation points: 92 ± 81 [mean ± SD]) participated in the present study. The athletes were informed about the measurement procedure and provided informed written consent before participation. The Regional Ethics Committee, Trondheim, Norway, approved this study.

### Ethics statement

The experimental procedures were approved by the Regional Ethics Committee for Medical and Health Research in Trondheim, and the protocol and procedures were verbally explained to each subject prior to obtaining written consent to participate.

### Skiing equipment

**Fig 1:**
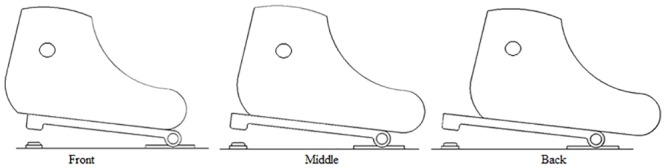
Illustration of the constructed ski binding. Boot positioning with respect to the hinge located at a front (first distal phalanx), middle (first proximal phalange) and rear (metatarsal-phalangeal joint) position (illustrated from left to right, respectively). The lever arm created between the ankle joint and the hinge is 100% at the front, and reduced by 15% and 30% for the middle and rear pivot positions, respectively.

A klapskate-style binding was designed to create a rigid soled ski boot. This binding allowed for a traditional skate boot to be mounted to the binding at three different anterior-posterior positions, ranging from the front of the first distal phalange, to the metatarsal-phalangeal joint (Fig 1). Skiers used a modified Madshus Nano-Carbon skate boot (Madshus/K2, Biri, Norway) that was custom fit to a prototype binding designed specifically for this project (IDT Sports, Lena, Norway). To exclude variations in rolling resistance, all skiers used the same pair of roller skis with standard wheels (IDT Skate, IDT Sports, Lena, Norway). Skiers used a pole with a modified rubber tip. The pole length was approximately 90% of the skier’s height.

### Measurement procedures

Upon their arrival to the laboratory, the athletes performed a low intensity 20-minute ski-specific warm up while roller ski-skating on the treadmill with their traditional shoe and binding systems. All hinge positions were investigated while roller skiing by employing the G3 (V2 or double-dance) ski-skating technique on a 5% inclined treadmill at 3.89 m/s. Speed and incline was chosen based on earlier testing which induces aerobic steady-state and was monitored with blood lactate and heart rate values. The order of the hinge positions was performed as a randomized controlled trial. Between each different hinge position, the athletes were given a 15-minute active recovery session and while doing so completed a questionnaire to characterize the system. Prior to each new hinge position, the athletes were given a 4-minute low intensity familiarization period. The specific speed, incline, and time were chosen in order to avoid muscular fatigue and induce an aerobic steady-state situation in order to properly assess gross efficiency.

### Instrumentation

Treadmill tests were performed on a 5x3m motor-driven treadmill (Forcelink B.V., Culemborg, The Netherlands). Incline and speed were calibrated using the Qualisys Pro Reflex system and Qualisys track manager software (Qualisys AB, Gothenburg, Sweden). VO_2_ was assessed by employing open-circuit indirect calorimetry with an Oxycon Pro apparatus (Jaeger GmbH.iam, Hoechberg, Germany). Prior to each measurement, the VO_2_ and VCO_2_ gas analyzers were calibrated against ambient air and commercial gas with known concentrations (16.00 ± 0.04% O_2_ and 5.00 ± 0.1% CO_2_, Riessner-Gase GmbH & Co, Lichtenfels, Germany). The inspiratory flow meter was calibrated with a 3L volume syringe (Hans Rudolph Inc., Kansas City, MO). Blood samples (20μl) from the fingertip were analyzed using the Biosen C-line Sport lactate assay system (EKF-diagnostic GmbH, Magdeburg, Germany).

Surface EMG signals were recorded for six lower limb muscles: semitendinosus (ST), rectus femoris (RF), vastus lateralis (VL), vastus medialis (VM), lateral gastrocnemius (MG), and tibialis anterior (TA). The athlete setup began with skin preparations, including shaving and cleaning the skin with alcohol, followed by the placement of bipolar, disposable pre-gelled Ag/AgCl surface electrodes with 20 mm inter-electrode distance (Noraxon dual electrodes, Noraxon USA Inc., Scottsdale, AZ). The placement of these electrodes followed the recommendations of the SENIAM (Surface Electromyography for the Non-Invasive Assessment of Muscles) project [15]. In addition, an accelerometer was placed on the rear end of the ski to determine the skating cycles. The EMG and acceleration data were sampled at 1500 Hz. The EMG signals were band-pass analog filtered at 10–500 Hz and sampled with a gain of 1000 using TeleMyo 900 (Noraxon USA, Inc., Scottsdale, AZ).

### Gross efficiency

Testing was performed at aerobic steady state submaximal intensity, which is required to calculate efficiency. Gross efficiency was calculated as the external work rate performed by the entire body, divided by the aerobic metabolic rate and presented as a percentage. Work rate was calculated as the sum of power against gravity (P_g_ = m · g · sin α · v) and friction (P_f_ = m · g · cos α · μ · v), where m is the body mass of the skier, g is the gravitational acceleration, α is the angle of treadmill incline, v is the speed of the treadmill belt, and μ is the frictional coefficient (.022). The rolling friction force (F_f_) of the skis was determined prior to the test by using a towing test, while the friction coefficient (μ) was calculated by dividing the friction force by the normal force (F_n_): μ = F_f_ · F_n_^-1^ [16]. The aerobic metabolic rate was calculated as the product of VO_2_ and the oxygen energetic equivalent using the associated respiratory exchange ratio and standard conversion tables [17]. After a 20-minute warm up on the treadmill, a steady rolling friction of the skis during the whole test was assumed [18].

### Analysis of cycle characteristics and electromyographic data

The moment of the ski takeoff was determined in the acceleration signal as the first point where the slope of the vertical ski acceleration exceeded 8 m/s^2^ for 4 ms. These points served as trigger points, marking the beginning and end of each skating cycle. For each trial, 30 skating cycles were extracted for further analysis. The average cycle length and cycle rate were calculated from these 30 cycles.

The EMG analysis was conducted using custom-written Matlab^™^ (The MathWorks Inc., Version R2014a, Natwick, MA, USA) codes and included the following steps: the EMG signal of each muscle was wavelet transformed [19] to obtain the intensity of the EMG signal. The wavelet transformation was based on 14 non-linearly scaled wavelets covering the whole frequency range between 7 and 250 Hz [19]. The total intensity was obtained as summation of the intensities in each frequency band. Then, 30 stride cycles with durations between 1.5 and 2.2 seconds were determined; cycles outside this range were deemed outliers and were thus omitted. The EMG intensity was resampled such that 501 data points represented each stride-cycle. All stride-cycle waveforms were normalized by setting the total intensity per cycle in each muscle to 1. Due to the normalization, information about the absolute magnitude of the muscle activity was lost, but the waveform shapes can be compared across muscles and between participants and hinge positions [20]. The EMG signal waveforms were taken for each muscle, cycle, athlete, and hinge position in order for the respected EMG peak intensity to be found. EMG peak intensity values were then compared across hinges for all subjects and within each muscle.

Additionally, all waveforms of the six muscles were concatenated into a 3006-dimensional vector. All vectors from the eleven participants, each skating with three different hinge positions, formed a 990 x 3006 matrix **M** (eleven participants * 30 cycles * 3 hinge positions * waveforms (501 points) of 6 muscles) which was submitted to a principal component analysis (PCA).

The PCA yielded: 1) a set of eigenvectors PC_k_; 2) the associated eigenvalues EV_k_; and 3) the principal component scores (PC-scores) ξ_k,i_. The indices k and i denote the order of the principal component and the trial number, respectively. The eigenvectors represent multi-muscle patterns of correlated deviations from the mean waveform, which are interpreted as muscle “synergies” [13, 21, 22]. The eigenvalues quantify how much variability was represented by each eigenvector. These values were normalized by expressing them as a percentage of the sum of all eigenvalues. The PC-scores (ξ_k,i_) are a measure of how similar the measured waveform i is to each principle component k. For each hinge position and subject, 30 ξ_k,i_ were obtained (from 30 stride cycles).

### Statistical analysis

All data were checked for normality using a Shapiro-Wilks test and were presented as mean and standard deviation (SD). The effects of hinge position on GE, kinematics (CL and CR), and EMG peak intensity for reach muscle were tested using a one-way ANOVA for repeated measures. The statistical analyses were performed using the IBM SPSS 21.0 for Windows (SPSS Inc., Chicago, IL). The level of statistical significance was set to α = 0.05 in all tests.

For the EMG-PC scores, two types of statistical analyses were conducted. Between-subject effects were determined by calculating the respective means of the 30 PC-scores obtained from the 30 cycles of each subject and each hinge position. Then the distribution of these subject means were compared between hinge conditions using the repeated measures ANOVAs. Intra-subject effects of the hinge position on the EMG-PC scores were analyzed by performing one-way ANOVAs on the 90 PC-scores obtained for each subject.

## Results

### Physiological responses and cycle characteristics

The hinge positioning did not significantly affect VO_2_, gross efficiency, heart rate or blood lactate concentration, nor was there any significant change in the cycle rate or cycle length across hinge positions (Table 1). For the comfort rating, most athletes (64%) ranked the middle hinge position highest (most comfortable), compared to 9% and 27% for the front and rear positions, respectively.

**Table 1:** Oxygen uptake, gross efficiency, cycle rate, heart rate, blood lactate, as well as the level of significance during 4-minute submaximal roller ski skating (5% incline at 14 km·h^-1^) across three different hinge positions (front, middle, rear) on a modified klapskate binding for 11 male elite cross-country skiers (mean ± SD).

	Front	Middle	Rear	Significance
VO_2_ (ml·kg^-1^·min^-1^)	50.2 ± 3.3	50.3 ± 2.1	49.7 ± 2.3	*P* = 0.54
Gross Efficiency (%)	16.6 ± 0.5	16.7 ± 0.5	17.1 ± 0.7	*P* = 0.12
Cycle Rate (Hz)	0.51 ± 0.02	0.51 ± 0.02	0.52 ± 0.02	*P* = 0.21
Heart Rate (bpm)	157 ± 11	157 ± 11	157 ± 10	*P = 0*.*79*
Blood Lactate (mmol L^-1^)	3.26 ± 0.10	2.97 ± 0.84	3.15 ± 1.32	*P = 0*.*45*

### Peak intensity

**Fig 2:**
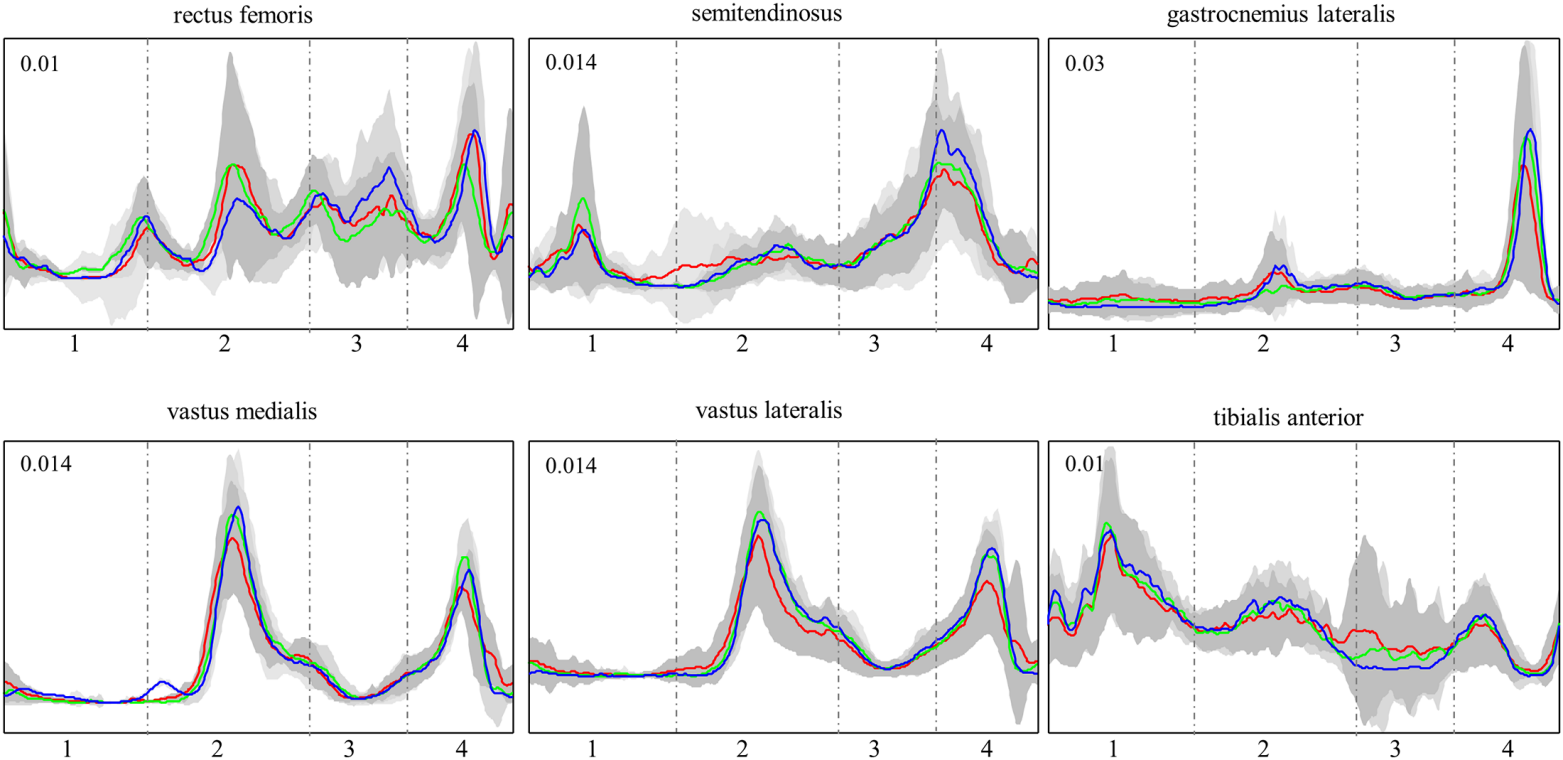
Mean EMG intensity and standard deviation of the 11 subjects’ for six lower limb muscles at each hinge position, front (red), middle (blue), and back (green). The skating cycle is represented from ski take-off to the following ski take-off. The vertical dashed lines separate phases: swing (1), ski-plant (2), pole-loading (3) and push-off (4). Each waveform has been scaled to its maximum range.

The peak intensity for the 11 subjects showed no significant change as an effect of hinge position across all six muscles (Fig 2).

### Muscle synergies

**Fig 3:**
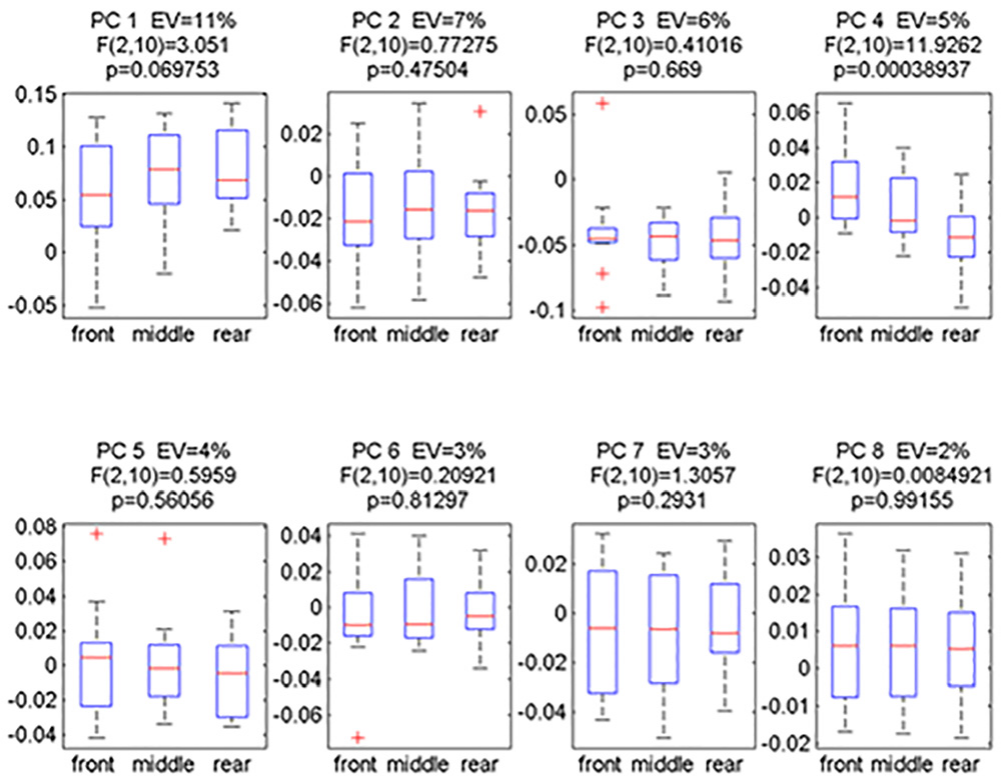
Boxplots for the first eight principal component score distributions of the 11 subjects’ mean scores obtained for each hinge position condition. The eigenvalues (EV) quantify how much of the total elecromyographic waveform variability that is represented by each specific PC (in %). The F and p values were calculated using a repeated measure ANOVA.

**Fig 4:**
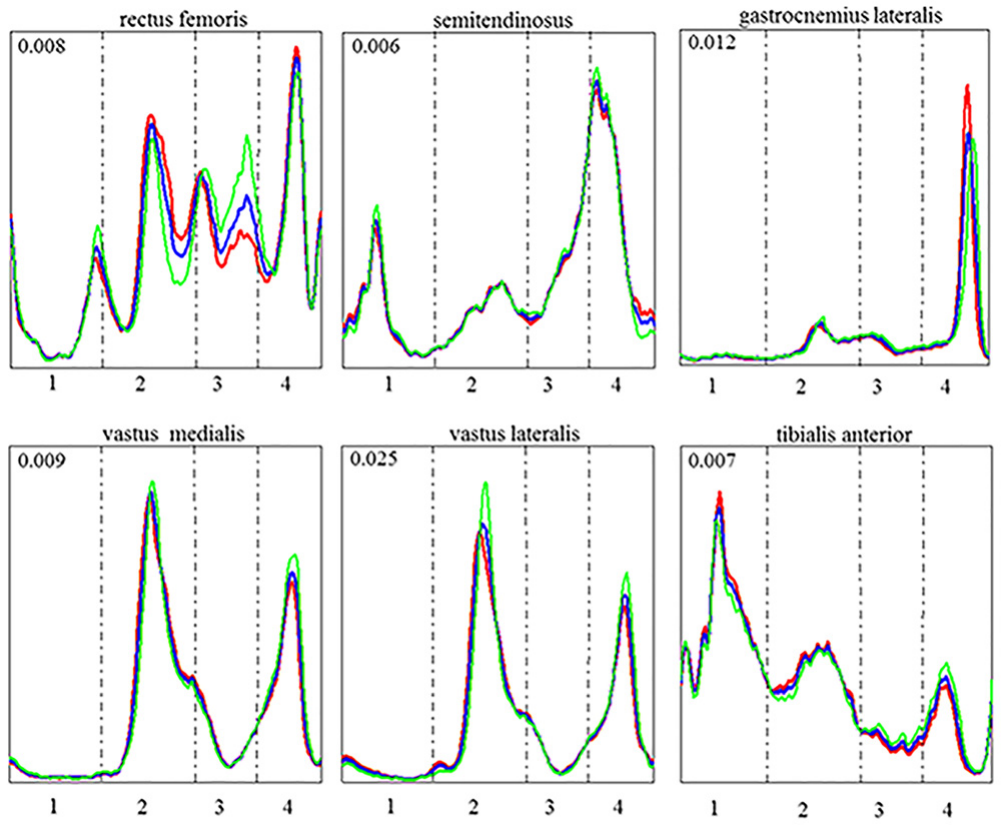
Visualization of the changes in elecromyographic waveforms quantified by principal component 4 (PC4). The waveforms were reconstructed for six lower limb muscles for each hinge, front (red), middle (blue), and back (green). The skating cycle is represented from ski take-off to the following ski take-off. The vertical dashed lines separate phases: swing (1), ski-plant (2), pole-loading (3) and push-off (4). Each waveform has been scaled to its maximum range.

Within the first eight PCs, only the scores of PC_4_ indicated significant inter-subject effects of the hinge positioning (Fig 3). Across all six muscles, the first four PCs together were responsible for 29% of the EMG waveform variance, with the fourth PC vector accounting for 5% of the total variance. Fig 4 shows a visualization of the correlated deviations from the mean EMG waveform that is quantified by the PC_4_ vector.

**Fig 5:**
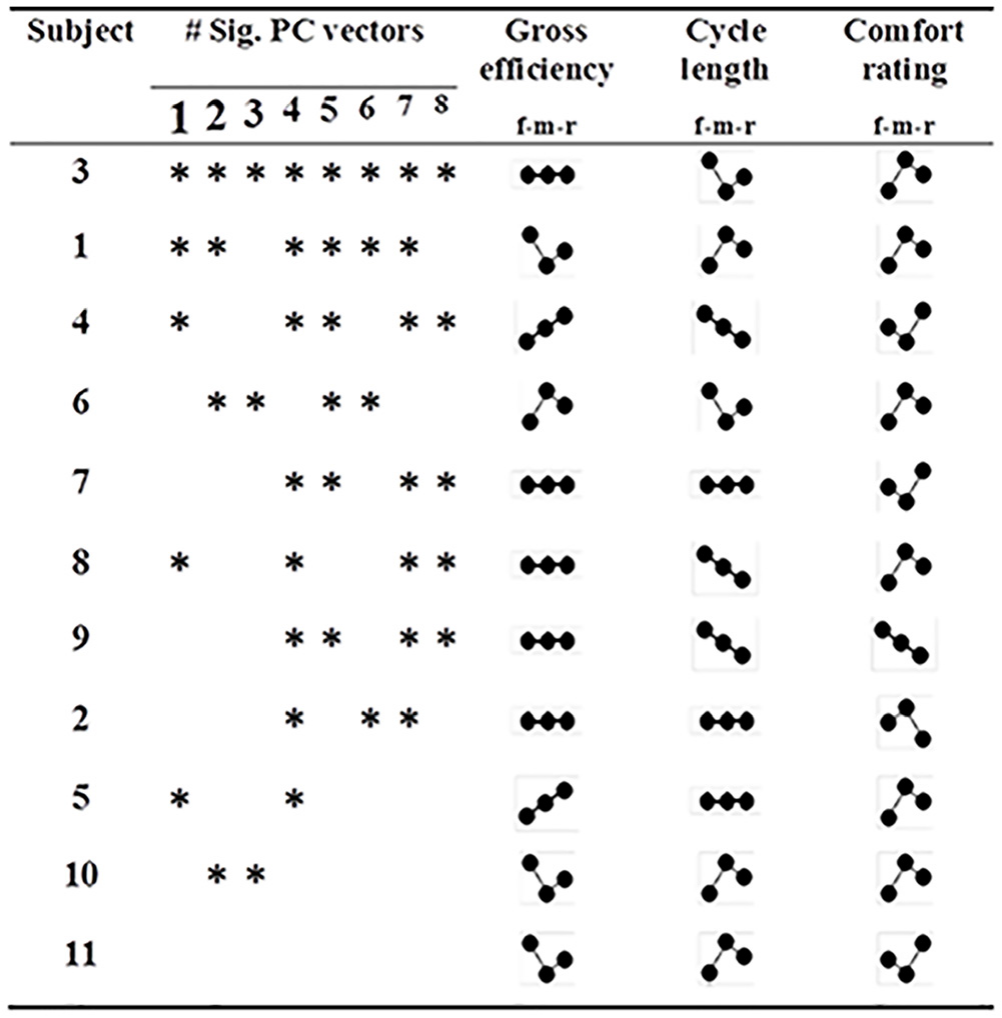
Illustration of the significant intra-subject effects of hinge position (indicated by *) in the first eight PC vectors displayed for all subjects, as well as the patterns of intra-individual changes in gross efficiency, cycle length, and comfort rating represented with respect to front, middle or rear (f-m-r) hinge position. Dotted lines represent the patterns of increasing (⋰), deceasing, (⋱), no change (⋯), and other variants of change (∴) between conditions.

The intra-subject assessment of hinge positioning for the first 8 synergies showed individual variations. The distribution of significant PC scores is shown in Fig 5. The intra-subject assessment for the number of significant PC scores showed no obvious relationship between efficiency, cycle kinematics or comfort rating.

## Discussion

The current study had two central outcomes. First, we could not confirm the underlying hypothesis that hinge positioning would systematically affect efficiency or cycle characteristics in ski-skating. Second, our EMG analysis suggested that systematic changes in the EMG activation patterns do occur, which accompanies changes in hinge positioning. However these changes occur on a synergistic level and not while comparing peak intensity within each muscle. Our study therefore fills a significant gap in the current understanding on the klapskate mechanism, since previous studies did not detect any adaptations in muscle activation, although such adaptations are highly plausible [1]. Differences in the muscle activation could be detected since the current study used a different analysis approach as compared to previous studies.

It is somewhat surprising that the current study could not verify changes in gross efficiency or cycle characteristics, since the underlying mechanisms that were discussed in the introduction are well-established in the literature [1, 4, 5]. Perhaps the main difference between our study and related previous studies is that upper body poling is used while ski-skating and is responsible for a significant fraction of the forward propulsion [23, 24]. During the submaximal skiing used here, skiers may have compensated for a restricted push-off by using upper body poling more extensively, such that gross efficiency or cycle characteristics did not change. However, it is equally likely that skiers may have chosen to ski with a fixed stroke frequency and maintain velocity by regulating work per stroke [5]. Thus, cycle characteristics would remain equal between hinges, while the muscle specific work may shift. In retrospect, our hypotheses may have been better tested in ski-skating without poles in order to provide improved cross-over with speed-skating results, or at a higher intensity. However, skating without poles would compromise the study’s external validity and gross efficiency is determined more reliably below the anaerobic threshold, and where fatigue is less likely to affect the measurement outcomes.

When comparing peak intensity across hinge positions within each muscle, it is not surprising that no differences were found either in previous klapskate research [1] or in the current study, due to high variability exhibited in EMG signals. This variability is not only a product of multifaceted internal and external influencing factors, it is also an inherent characteristic of the neuro-muscular system [25]. EMG variability is functionally important, for example, for fatigue prevention [21] or adaptability [26]. A change in equipment (i.e., hinge positioning) adds one more source of EMG variability. The extent of equipment-related variability might be small compared to the variability produced by other sources; however, it should manifest as a specific multi-muscle pattern that relates to the functional changes produced by the adjustment in equipment. Based on the consideration of EMG exhibiting high variability, PCA was used as an additional method for analysis of EMG signals. This analysis method detects patterns of correlated variations in waveforms [12] and was therefore employed in the current study to determine if between-subject or within-subject adaptations take place in muscle activation during submaximal roller skiing. Since this study did not find differences in efficiency or cycle characteristics, it is not surprising that the pattern in which systematic changes were found between-participants (PC_4_) was responsible for only 5% of the total variability in muscle activation.

Several features represented by PC_4_ (Fig 4) are in line with the effects that would be expected based on the previously described mechanism explaining the biomechanical effects of hinge positioning: placing the hinge in the front position creates a longer lever arm with respect to the ankle joint. During push-off one would therefore expect higher muscle activation in the gastrocnemius and rectus femoris, but lower activation in the vasti muscles [1, 5]. In the swing phase, one would expect higher muscle activation in the tibialis anterior due to the longer lever arm used to lift the ski off the ground [27]. The characteristics that PC_4_ exhibits during the before-mentioned phases in these three muscles agree well with the expected pattern. Furthermore, our results also suggest that by changing hinge position, other muscle coordination adaptations may occur throughout various phases of the ski-cycle that have not previously been described. For example, relative to the activation during other cycle phases, an increase in rectus femoris and a decrease in the vasti muscle activation seem to occur after ski plant and early in the gliding phase in PC_4_. This suggests differences in the stabilization while the contralateral leg finalizes the push-off. Furthermore, significant differences were observed in PC_4_ during the loading phase for the rectus femoris, which may suggest variations in the ability to position the body prior to pole loading.

The intra-subject analysis revealed multifaceted individual responses among participants to the change in hinge position. Previous studies already suggested that skiers’ individual responses are influenced by many interacting factors besides hinge position, such as muscle strength, dynamic range of motion, or the unique physique of each athlete [28]. Our results are suggestive of numerous degrees of freedom (DOFs) involved with whole-body movement, thereby allowing for highly individualized task solutions. Large DOFs allow each athlete to individually ensure both unique stability of the movement and flexibility of patterns when dealing with hinge position. Furthermore, each athlete’s specific coordinative solution is affected by unpredictable factors, such as variation in the internal states of the body and their unique response to the external stimuli.

Limitations: the analysis applied in the current study first split the EMG signals into components (PCA) where the deviations from the mean EMG waveform are correlated in a specific pattern; and then tested for statistical differences when the individual waveforms are projected onto these patterns. Consequently, if a significant difference is observed, as in PC_4_, then this indicates that the pattern as a whole changes when changing the hinge position. Features of PC_4_ can be discussed, but statistical differences cannot be pinpointed directly to specific muscles or specific points within the skating cycle. Another issue to consider is that normalization is necessary when comparing synergistic EMG waveforms between participants. The current study normalized the waveforms such that each muscle contributed the same amount of variability to the PCA input. Other normalizations are possible and may yield different results. Hence, while our study proves that differences in muscle activation exist, the differences we described here account for only 5% of the total EMG waveform variance and may not be the only ones that take place. Furthermore, it is likely that the coordinated structures associated with hinge position are likely to change with varied intensity. Another limitation of the current study is that lower limb kinematics was not determined and that the relationship between changes in muscle synergies and possible changes in joint kinematics were not investigated.

## Conclusions

The current results did not confirm the hypothesis that hinge positioning would systematically affect gross efficiency or cycle characteristics while roller ski-skating. However, the analysis of EMG signals revealed a small but significant effect of hinge positioning on muscular coordination. The pattern of change in muscle activation agrees with previously described mechanisms that explain the effects of hinge positioning in speed-skating. Finally, the within-subject results in the EMG analysis suggest that in addition to the effects seen between-participants, there appears to be numerous additional muscular coordination adaptations of various form employed by some, but not all participants.
